# Understory dominance and the new climax: Impacts of Japanese knotweed (*Fallopia japonica*) invasion on native plant diversity and recruitment in a riparian woodland

**DOI:** 10.3897/BDJ.5.e20577

**Published:** 2017-11-13

**Authors:** Matthew J. Wilson, Anna E. Freundlich, Christopher T. Martine

**Affiliations:** 1 Friends of the Verde River, Cottonwood, AZ, United States of America; 2 University of Northern Colorado, Greeley, CO, United States of America; 3 Bucknell University, Lewisburg, PA, United States of America

**Keywords:** biodiversity, *Polygonum cuspidatum*, recruitment, *Reynoutria japonica*, riparian, undergraduate research

## Abstract

Riparian forests exhibit levels of ecological disturbance that leave them especially prone to biological invasions. Japanese knotweed (*Fallopia japonica*) is particularly suited to these habitats and is an aggressive invader along watercourses throughout its now-global range as an exotic invader. Using one of the few Silver Maple Floodplain Forest communities that has not been invaded by *F. japonica* in the West Branch Susquehanna River valley (Pennsylvania, USA) as a baseline, this study examines whether and how this primarily intact riparian forest community differs from nearby invaded communities in terms of 1) native species richness, 2) native species density, and 3) riparian forest tree recruitment. Defining a baseline (intact) community composition will inform restoration plans for local riparian forests where knotweed might be eradicated or reduced. Invaded and non-invaded sites differed statistically across species richness, species density, and tree recruitment. Our results suggest that *F. japonica* has reduced the diversity and abundance of native understory riparian plant species. The species also appears to have suppressed long-term tree recruitment, setting up a trajectory whereby the eventual decline of trees currently in the canopy could shift this community from a tree-dominated riparian forest to a knotweed-dominated herbaceous shrubland.

## Introduction

Invasive species are known to cause community level effects that include reductions in richness, density, and recruitment of native flora and fauna. This can be especially true for habitats in which dynamism and disturbance, characteristics invasive taxa are often adapted to, are natural elements of ecological cycles (e.g. Rejmanek and Richardson 1996). Large-river riparian systems typically support diverse plant communities well-adapted to the disturbance present in these dynamic and high-energy environments such as flooding, loss of substrate by erosion, and deposition of bank materials (Naiman and Décamps 1997) and are also important in sustaining local biodiversity. As the transition-zone between aquatic and terrestrial habitats these areas provide habitats unique to surrounding areas and are frequently disturbed, which contributes to the elevated levels of biodiversity in these communities and also makes them prone to invasion (Planty-Tabacchi et al. 1996). The ecological services provided by riparian habitats are strongly linked to the vegetation present, with the effects of invasive species on native plant communities of particular concern (Herrera and Dudley 2003, Hood and Naiman 2000).

Although some sexual reproduction occurs in invasive populations of *F. japonica* (Forman and Kesseli 2003, Bram and McNair 2004, Grimsby et al. 2007, Wymer et al. 2007), the species spreads mainly through rhizomes (Pyšek et al. 2003, Bram and McNair 2004), a dispersal habit leading frequently to invasions along rivers, streams, roadways, and other edge habitats where disturbance levels are high (Barney et al. 2006). Once established, *F. japonica* forms dense thickets that can be up to three meters tall, affecting light levels that reach the forest floor and reducing photosynthetic production by co-occurring plants (Siemens and Blossey 2007, Urgenson et al. 2014). Considerable generation of stem litter after each growing season (Topp et al. 2007, Urgenson et al. 2009), release of allelopathic compounds (Murrell et al. 2010, Pyšek et al. 2011), impacts on soil microbes (Siemens and Blossey 2007), and homogenization of soil nutrient profiles (Dassonville et al. 2007) may act as barriers to germination that reduce recruitment of native plants.

We conducted this study along the West Branch of the Susquehanna River (Lewisburg, Pennsylvania, USA), where non-native Japanese knotweed (*Fallopia japonica* (Houtt.) Ronse Decr.; Synonym: *Polygonum cuspidatum*, *Reynoutria japonica*) is the dominant understory component in the majority of associated Silver Maple Floodplain Forests (SMFF). While this forest type is not rare in Pennsylvania, it has been identified as a community of conservation concern because large contiguous SMFFs are uncommon and increasingly lost to development and agriculture, impacted by changing flood regimes, or invaded by aggressive exotic plants that displace native ones (Zimmerman 2011). Local consequences of knotweed dominance in particular have not been explored but studies of *F. japonica* (and its closely-allied invasive congeners, *F. sachalinensis* (F. Schmidt) Ronse Decr. and F. × bohemica (Chrtek & Chrtková) J.P. Bailey in other river systems have reported declines in native plant species in riparian zones (Barney et al. 2006, Gerber et al. 2008, Urgenson et al. 2009) with consequences including invertebrate communities (Kappes et al. 2007, Gerber et al. 2008) and their predators (Maerz et al. 2005), litter nutrient quality (Urgenson et al. 2009), and aquatic food webs (Lecerf et al. 2007).

Zimmerman (2011) suggested Silver Maple Floodplain Forests in Pennsylvania require further study because a) Localized ecoregional variants of this widespread community type need to be surveyed and defined and b) Few high-quality SMFF examples remain on the landscape. The purpose of this study was to compare an unusually intact SMFF along the West Branch of the Susquehanna River to a nearby SMFF heavily invaded by *F. japonica*. The two primary goals were to define a baseline species composition for an intact (and localized) example of this increasingly threatened riparian community type and to then assess the ongoing impact of knotweed invasion on this assemblage of taxa. Community data were collected on opposite riverbanks to address whether SMFF communities not invaded by *F. japonica* differ from invaded (dominated by *F. japonica*) SMFF communities in terms of 1) native species richness, 2) native species density, and 3) riparian forest tree recruitment. Based on our results, we hypothesize the dominance of *F. japonica* has reduced the diversity and abundance of native understory riparian plant species and led to reductions in riparian forest tree recruitment in the Silver Maple Floodplain Forests of the Susquehanna Valley.

## Methods

### Study Area

This study was conducted in the riparian zone of the West Branch Susquehanna River in Lewisburg, PA (40°57'18"N, 76°52'39"W), a 250-300 m wide 7th order river with average discharge of 308 m^3^/s at our study site in Lewisburg, Pennsylvania, USA (USGS 2016). The local riparian habitat supports a plant community meeting the definition of a Silver Maple Floodplain Forest (Fike 1999, Zimmerman 2011) with an overstory consisting of *Acer saccharinum* L. (silver maple, Sapindaceae) as a dominant component typically accompanied by *Betula nigra* L. (river birch, Betulaceae), *Platanus occidentalis* L. (sycamore, Platanaceae), and *Acer negundo* L. (boxelder maple, Sapindaceae). The study location was selected because of the presence of a relatively intact (native-dominated) plant understory community mirrored by a knotweed-infested understory community on the opposite bank of the same river reach.

### Vegetation Sampling

Each study area was divided into a set of 30x15 m (=450 m^2^) rectangular plots run along 400 m transects (separated along transects by 30 m lengths), with the long sides of the plot parallel to the riverbank and the shorter sides running perpendicular to the riverbank. Forty-four 450 m^2^ ‘large plots’ were sampled between the two bank communities. Within each large plot, vegetation was sampled across the herb and canopy layers during the 2013 field season (May-July). At the bisector of each large plot, running perpendicular to the bank, stem counts were conducted within 0.25 m^2^ square plots at the 2 m, 7 m, and 12 m marks (where 0m occurred closest to the water’s edge). Plants were considered to be within the herb-layer if they were less than 0.5 m tall. Follow-up observations were conducted in the subsequent fall and spring seasons to establish presence/absence for all understory species encountered within the 30x15 large plots, including taxa that were not captured in the 0.25 m^2^ plots.

The canopy layer was surveyed from the midpoint of each 30x15m large plot within a circle plot of 5 m radius (80 m^2^ survey area). Plants were counted as canopy trees if they were measured to have a diameter at breast height (dbh) of at least 10 cm and had any portion of stem within the circle plot. For each of these trees, dbh was recorded and used as a rough proxy for age as tree cores proved problematic because silver maples typically hollowed with age. All individuals across vegetation layers were identified to species using Rhoads and Block (2007), recorded, and vouchered in the Wayne E. Manning Herbarium (BUPL) at Bucknell University. All data used for this manuscript have been deposited in the Knowledge Network for Biocomplexity (https://knb.ecoinformatics.org; Wilson et al. 2017).

### Data Analysis

To investigate the effects of *F. japonica* on overall patterns in community composition between 0.25 m^2^ herbaceous samples we used Principal Component Analysis (PCA) in the R package vegan (version 2.0-10; Oksanen et al. 2013). To test for significant difference in community composition between invaded and non-invaded communities (i.e. native- and knotweed-dominated understory communities) we generated 95% confidence intervals for sample PCA scores along Axis 1 and Axis 2, grouped by bank (ellipse package; Murdoch and Chow 2013). In addition to overall patterns in community composition, we tested whether high densities of *F. japonica* affected the density of native species, invasive species, average species richness, and density by species of herbaceous taxa by comparing samples by bank with a nonparametric Mann–Whitney U test and Bonferroni correction for multiple comparisons. To test if *F. japonica* also affected tree species we used Kruskal–Wallis non-parametric ANOVA by ranks to compare tree density between banks. We also generated histograms of tree size (dbh) as an indirect measure of recruitment and age class compositional differences between invaded and non-invaded communities. All analyses were performed in the R statistical environment (R Core Team 2013) with figures generated in the ggplot2 package (Wickham 2009). We also investigated within-bank beta-diversity as a measure of homogenizing effects of *F. japonica* on community composition using the multivariate approach of betadisper (vegan package) with ANOVA of group median centroids to test significance.

## Results

Overall, our 0.25 m^2^ plots captured 32 (6 non-native and 26 native) of 80 herb-layer species observed by presence/absence, with only 12 species occurring in both non-invaded and invaded plots (Table 1). Canopy surveys (80 m^2^) captured all eight observed tree species by presence/absence. In the herbaceous layer stem densities ranged widely from 576 stems/m^2^ to a minimum of 8 stems/m^2^. Non-invaded communities were dominated by native *Impatiens pallida* Nutt. (yellow touch-me-not, Balsaminaceae, 15/m^2^), *Toxicodendron radicans* (L.) Kuntze (poison-ivy, Anacardiaceae, 13/m^2^), and *Pilea pumila* (L.) A. Gray (clearweed, Urticaceae, 12/m^2^). After *F. japonica* (12/m^2^) the most common species in invaded communities were *T. radicans* (2.7/m^2^) and *I. pallida* (2.5/m^2^). In addition, the only native species with higher densities in invaded communities were *Arisaema dracontium* (L.) Schott (green dragon, Araceae, 0.10 versus 2.1/m^2^), *Polygonatum biflorum* (Walter) Elliott (Solomon’s-seal, Ruscaceae, 1.3 versus 1.8/m^2^), and *Matteuccia struthiopteris* (L.) Tod. (ostrich fern, Polypodiaceae, 0 versus 0.099/m^2^). Overstory surveys were dominated by *Acer saccharinum* (0.017/m^2^) in both non-invaded and invaded plots (Table 1).

**Figure 1.**
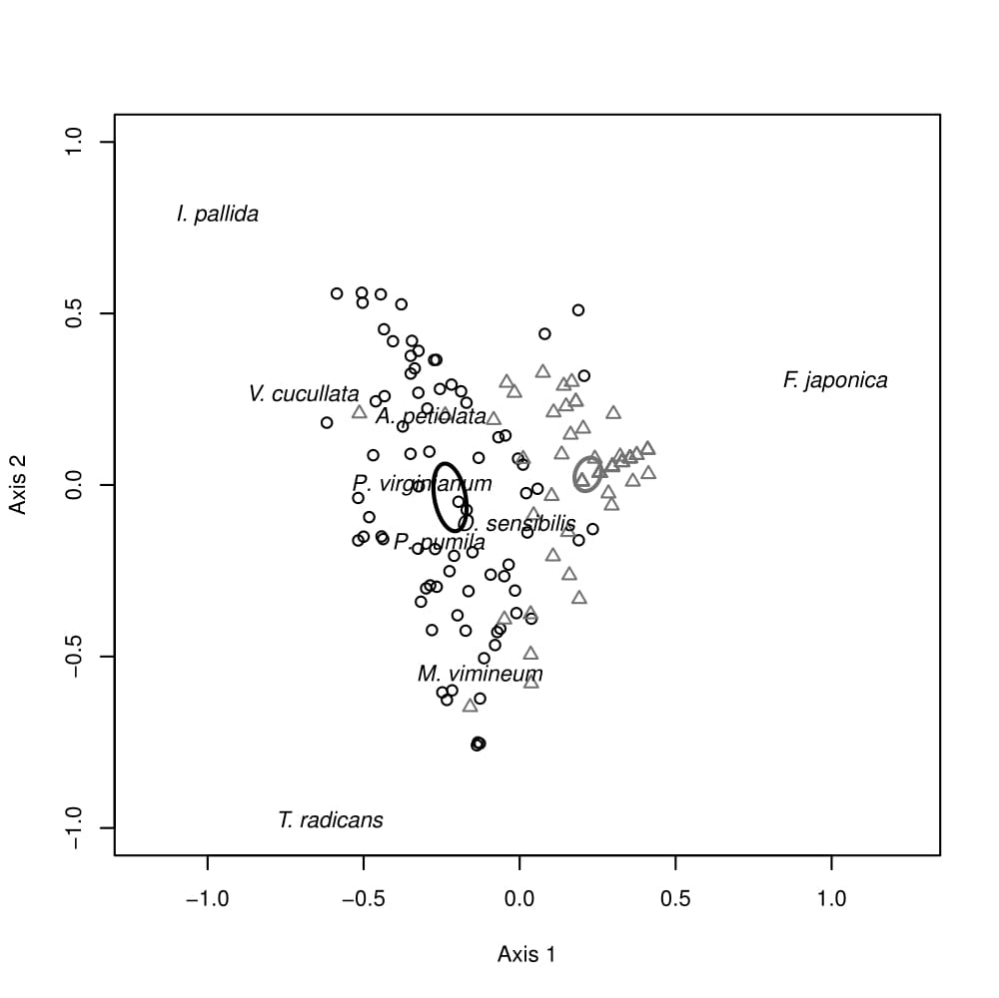
Principal Component Analysis (PCA) of herbaceous samples with 95% CI ellipses surrounding the centroid of each site type (invaded or non-invaded). Circles represent samples collected from the non-invaded Silver Maple Floodplain Forest site and triangles represent the site invaded by *F. japonica*, with strong species drivers of Axis 1 and 2 overlaid.

Our PCA results showed sampled communities cluster tightly by invaded and non-invaded river banks, with clear separation of 95% confidence ellipses for the centroid of each bank (Fig. 1). This pattern is strongly driven along Axis 1 by stem density of *F. japonica* (r = 0.84) in the invaded bank as well as *I. pallida* (r = -0.70), *Viola cucullata* Aiton (marsh blue violet, Violaceae, r = -0.65), and *T. radicans* (r = -0.45) in the non-invaded bank. Along Axis 2 the separation is primarily driven by *T. radicans* (r = -0.72), *I. pallida* (r = 0.56), and *Microstegium vimineum* (Trin.) A. Camus (Japanese stiltgrass, Poaceae, r = -0.47). While confidence ellipses show strong separation along Axis 1 this is not the case along Axis 2, indicating the separation along Axis 2 is unrelated to the river bank sampled.

**Figure 2.**
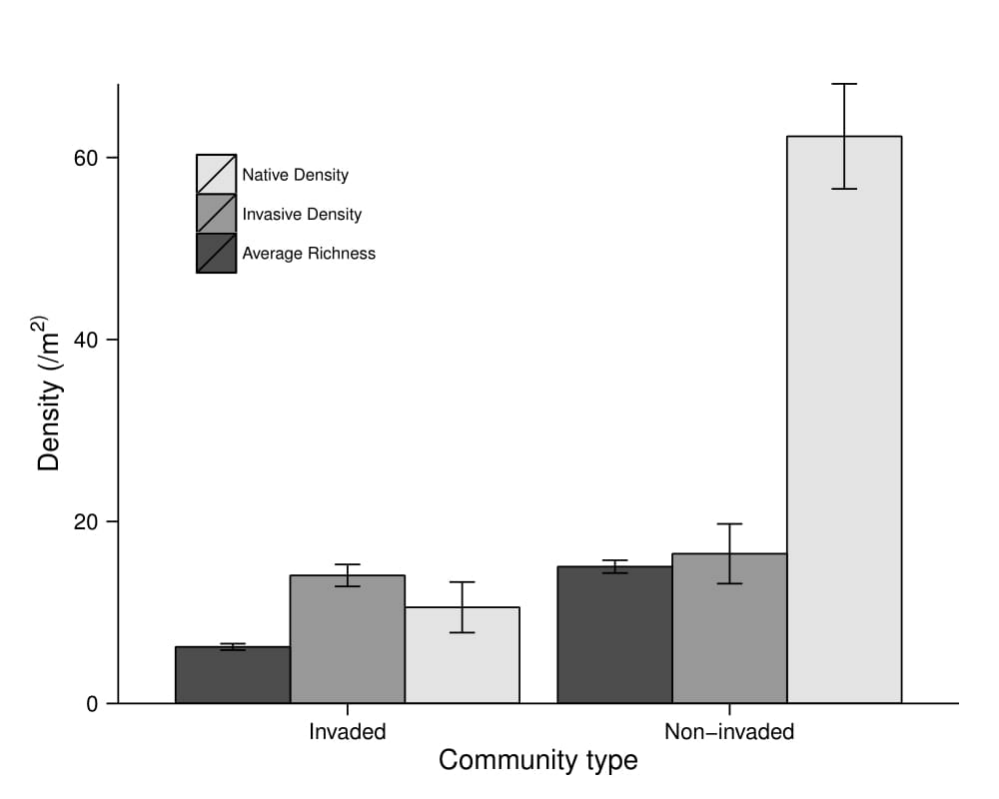
Bar plot of native species density, invasive species density, and average richness per sample by site type (invaded or non-invaded). Bars represent mean values ± standard error.

**Figure 3.**
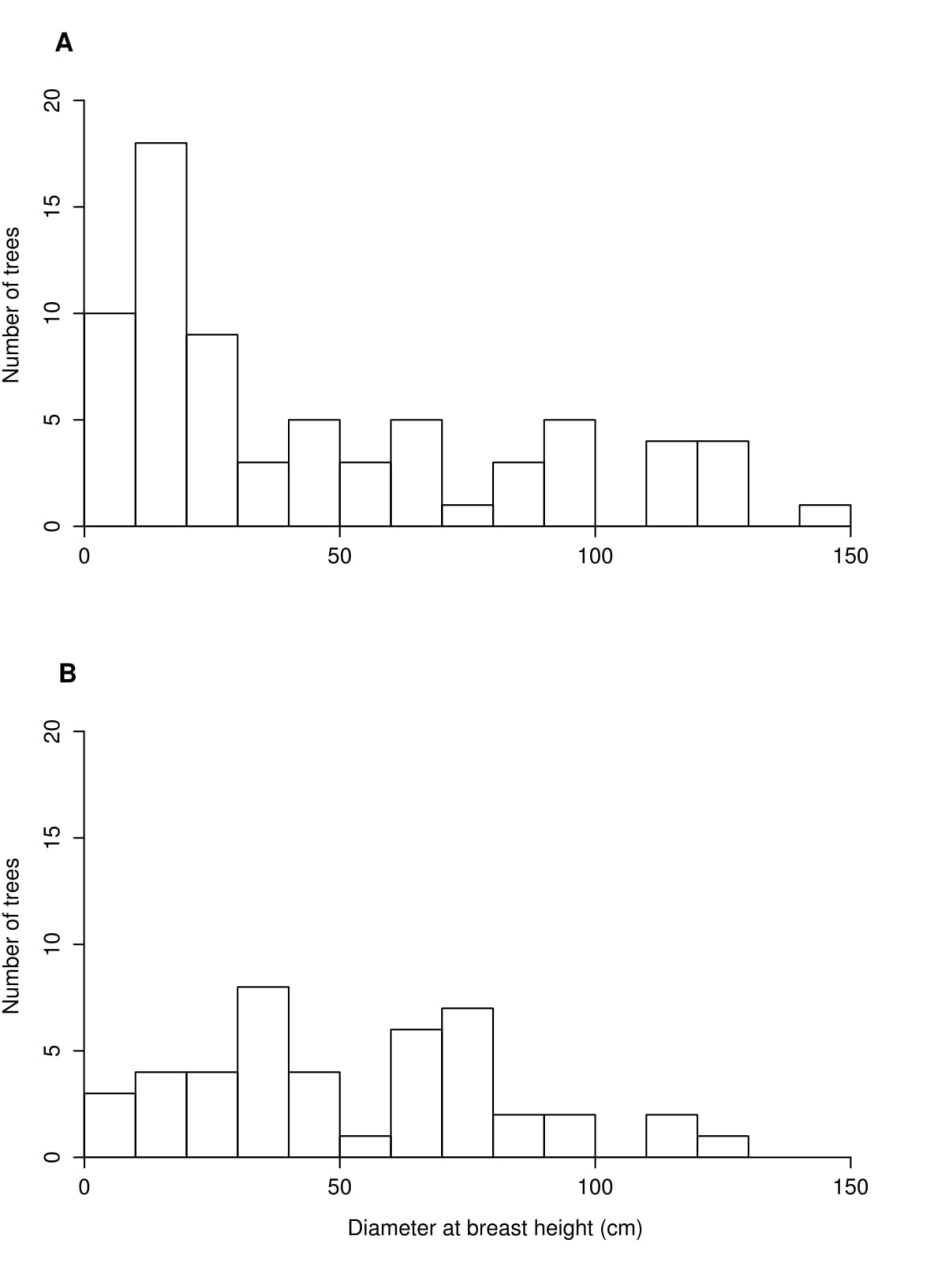
Number of canopy trees sampled in the (A) non-invaded forest and (B) invaded forest by their diameter at breast height. Counts are given in 10 cm increments for visual clarity.

For the non-invaded bank community, we found significantly higher native plant density (p < 0.0001), higher average richness per square meter (p < 0.0001), and higher exotic/invasive plant density (p = 0.015; Fig. 2). However, we are not confident in the difference in exotic/invasive plant density between banks as the distribution for the non-invaded bank is strongly bimodal, with a median of 8 plants/m^2^ while the invaded bank has a median of 12 plants/m^2^ as well as similar means. In addition, samples taken from the non-invaded bank showed significantly higher beta-diversity (i.e. community heterogeneity) than samples taken from the invaded bank (mean distance to group median centroids of 0.58 versus 0.37; p < 0.0001). Tree density was also significantly higher in the absence of knotweed (p = 0.021), a pattern that appears to be driven by lower tree recruitment in the presence of knotweed (Fig. 3).

## Discussion

Our results showed significantly lower native plant density, species diversity, and tree recruitment in the presence of *F. japonica*. Although we do not know what pre-invasion conditions were like in our study sites, we can infer the three differences noted above may be connected to the life history of *F. japonica* given the stark contrast in the abundance of this aggressive invader. Whether or not *F. japonica* is less of a driver of species diversity than a passenger (i.e. MacDougall and Turkington 2005) on some other ecological gradient (such as flooding frequency) in our sites is uncertain. However, Silver Maple Floodplain Forests of the Susquehanna River corridor clearly exhibit low species diversity across canopy levels when invaded by *F. japonica*.

Local decreases in native species density and richness between invaded and non-invaded SMFFs translated in our study to a loss of beta diversity in the herbaceous layer at the between-patch scale. The homogenization of local communities reduced overall densities of native species and may translate into localized extirpations. This homogenization is also evident in our PCA results, as the spread of samples collected from the non-invaded community is larger than that of the invaded community. While not a significant finding in itself, it is noteworthy that this increased spread (i.e. beta diversity) is visible primarily along Axis 2, the axis which was not strongly influenced by whether samples were collected in the invaded or non-invaded community type. Instead, our data suggest this high heterogeneity is at least partly driven by a tradeoff in dominance between *Impatiens pallida* and *Toxicodendron radicans* in non-invaded communities. In these samples, when *I. pallida* was present (59% of samples) it had a density of 26/m^2^ while the density of *T. radicans* in these samples was 9.4/m^2^. Conversely, in plots where *T. radicans* was present (48% of samples) it had a density of 26/m^2^ while the density of *I. pallida* was 11/m^2^. In addition, non-invaded sites exhibited patchy distributions of other abundant species such as *Viola cucullata*, *Pilea pumila*, and *Persicaria virginiana* (L.) Gaertn. contributing to the higher beta diversity between samples in the non-invaded community type.

While nearly all of the native understory species observed on both banks had lower densities in invaded plots (Table 1), three native herbaceous species appeared to do just as well on either side. The apparent success of *Arisaema dracontium*, *Matteucia struthiopteris*, and *Polygonatum biflorum* likely has less to do with ecological tolerance than it does with life history strategy. As long-lived species re-growing annually from perennial rootstocks (Bierzychudek 1982, Kenkel 1997, Levine and Feller 2004), these species can maintain their status in the understory community even if recruitment of new individuals is suppressed. In that sense, the occurrence of the three species in knotweed-dominated SMFF plots is likely relictual and reflects a pre-invasion history.

Similarly, our finding that younger age classes of trees are significantly less prominent in invaded sites allows us to infer that Japanese knotweed dominance might suppress tree recruitment in our study area, a potential threat previously hypothesized by PA Natural Heritage Program botanists for the SMFF community type (Zimmerman 2011). This is further supported by a general dearth of saplings and small trees in the invaded sites. Anecdotal reports from volunteers in nearby Milton State Park, a riparian island, claim little to no *F. japonica* occurred in local riparian forest until the early 1960s – an observation that corresponds temporally with the expansion of the species throughout northeastern North America noted by Locandro (1973).

The combination of limited tree and native herbaceous species recruitment in local habitats dominated by *F. japonica* suggests a potential shift in the ecological trajectory of local SMFF sites and a pathway to a new climax. If the observed patterns and the decline of the relict overstory continue in the coming decades, trees will not be replaced – eventually resulting in a climax community in which the canopy might consist of 2-3 meter tall Japanese knotweed thickets functioning as an herbaceous shrubland. As our data are limited to a small section of the Susquehanna Valley, more work is needed to investigate the patterns documented here and the hypothesized mechanisms behind them.

We suggest the maintenance of SMFF diversity in the study region is now likely reliant on conservation management practices, including removal of Japanese knotweed. Ongoing studies on the management of *F. japonica* in Pennsylvania (Gover A et al. 2005, Gover A et al. 2008) have found eradication depends upon a multiple-step Integrated Vegetation Management (IVM) approach that includes control and removal of the rhizome system. Given the prevalence of the species and its propagules throughout the region, any eradication program also necessitates long-term follow-up, removal of new recruits, and efforts to restore and/or reestablish native plant communities (Urgenson et al. 2014). Likewise, invasions of SMFFs that are not already dominated by *F. japonica* should be prevented by early detection and rapid removal of new propagules/colonists (e.g. Colleran and Goodall 2014, Colleran and Goodall 2015). In addition to *F. japonica*, our results suggest these efforts should account for other invasive species that have become prominent in the region, particularly *Alliaria petiolata* (M. Bieb.) Cavara & Grande (garlic mustard, Brassicaceae), *Microstegium vimineum* (Japanese stilt-grass), and *Persicaria perfoliata* (L.) H. Gross (mile-a-minute vine, Polygonaceae). Although the latter species was not encountered in our invaded plot sampling, it co-occurs with and grows upon *F. japonica* in other SMFF communities in the Susquehanna watershed (Martine pers. obs.).

One potential solution to diversity decline in regional SMFF communities is to encourage the growth of native woody cover in riparian zones. This may include *Toxicodendron radicans* (poison-ivy), a native (though often maligned) species that trades dominance (along with *Impatiens pallida*) with *F. japonica* in our sites. Even when dominant, *T. radicans* tends not to choke out native herbaceous species and appears to act as a nursery species for other native riparian forest species.

Our results suggest *F. japonica* has reduced the diversity and abundance of native understory plant species in one of the few intact Silver Maple Floodplain Forests on the West Branch Susquehanna River. The invader also appears to have suppressed long-term tree recruitment, with the potential to shift this SMFF community from a tree-dominated riparian forest to a knotweed-dominated herbaceous shrubland. While the findings of this study apply most directly to our local stretch of the Susquehanna River, they are generally translatable across SMFF communities in Pennsylvania and, given conservation concerns about this community type (Zimmerman 2011), may serve as a model and comparison for additional surveys in other parts of the state and beyond. Likewise, diversity survey results from our intact study site will now provide pertinent ecoregional data for a redefinition of the SMFF community type as part of current Natural Heritage Program efforts to revise statewide plant community designations (Ephraim Zimmerman, pers. comm.). Any future attempts at SMFF community restoration will depend on a clear definition of what pre-invasion communities may have looked like.

## Figures and Tables

**Table 1.** Densities of stems in 0.25 m^2^ herbaceous-layer plots by species and river bank. (I) denotes invasive or non-native species and (N) denotes native. Bolded lines indicate species with higher densities in the presence of Japanese knotweed. Significant differences are noted as: *** = p < 0.001, ** = p < .01, * = p < 0.05.

**Species Name**	**Non-invaded Bank**	**Invaded Bank**
*Alliaria petiolata* (I) ***	4.7	0.049
*Cerastium fontanum* (I)	0.81	0
*Hesperis matronalis* (I)	0.15	0
*Microstegium vimineum* (I)	9.0	1.7
*Persicaria perfoliata* (I)	0.10	0
***Fallopia japonica* (I)** ***	**1.7**	**12**
*Acer saccharinum* (N)	0.050	0.049
***Arisaema dracontium* (N)**	**0.10**	**2.1**
*Arisaema triphyllum* (N)	0.51	0
*Asclepias incarnata* (N)	0.25	0
*Asclepias syriaca* (N)	0.20	0
*Boehmeria cylindrica* (N)	0.15	0.049
*Cryptotaenia canadensis* (N)	0.10	0
*Eupatorium fistulosum* (N)	0.051	0
*Galium asprellum* (N)	0.46	0
*Geum canadense* (N)	0.20	0
*Impatiens pallida* (N) ***	15	2.5
*Laportea canadensis* (N)	0.96	0
*Lilium superbum* (N)	0.20	0.049
***Matteuccia struthiopteris* (N)**	**0**	**0.099**
*Onoclea sensibilis* (N)	1.1	0
*Parthenocissus quinquefolia* (N) **	3.6	0
*Persicaria virginiana* (N) ***	4.6	0.25
*Pilea pumila* (N)	12	0.25
***Polygonatum biflorum* (N)**	**1.3**	**1.8**
*Sicyos angulatus* (N)	0.051	0
*Thalictrum pubescens* (N)	0.10	0
*Toxicodendron radicans* (N) ***	13	2.7
*Ulmus americana* (N)	0.051	0
*Verbesina alternifolia* (N) *	1.1	0
*Viola cucullata* (N) ***	7.6	0.74
*Vitis* spp. (N)	0.20	0
